# World Health Organization Perspective on Implementation of International Health Regulations

**DOI:** 10.3201/eid1807.120395

**Published:** 2012-07

**Authors:** Maxwell Charles Hardiman, World Health Organization Department of Global Capacities, Alert and Response

**Affiliations:** World Health Organization, Geneva, Switzerland

**Keywords:** World Health Organization, international cooperation, regulations, international health regulations, WHO, implementation, disease, spread, pandemic, influenza

## Abstract

In 2005, the International Health Regulations were adopted at the 58th World Health Assembly; in June 2007, they were entered into force for most countries. In 2012, the world is approaching a major 5-year milestone in the global commitment to ensure national capacities to identify, investigate, assess, and respond to public health events. In the past 5 years, existing programs have been boosted and some new activities relating to International Health Regulations provisions have been successfully established. The lessons and experience of the past 5 years need to be drawn upon to provide improved direction for the future.

## Abstract 2

The regulations have substantially helped prevent and control the international spread of diseases, but their full potential has yet to be realized.

Throughout the >60 years that the World Health Organization (WHO) has been in existence, member states have made use of the constitutional provision that permits the Health Assembly to adopt regulations concerning sanitary and quarantine requirements and other procedures designed to prevent the international spread of disease (*1*). In 1951, the first such regulations, the International Sanitary Regulations, were adopted and focused on 6 communicable diseases requiring coordinated international measures to control their transmission between countries (*2*). By the 1990s, they had been amended and renamed the International Health Regulations (IHR); their application was reduced to only 3 diseases, and they were considered inadequate for addressing the increasingly globalized nature of health risks. In 1995, the Health Assembly called on the WHO secretariat to develop revised regulations that were more relevant to worldwide public health challenges (*3**–**5*). A process of intensive and wide technical consultation was followed by a series of intergovernmental negotiations in which WHO member states took control of the draft and negotiated additions and amendments to every aspect before agreeing to a final version in time for it to be adopted at the 58th Session of the Health Assembly (*6*).

Since entering into force in 2007, the IHR have provided a legally binding global framework to support national and international programs and activities aimed at preventing, protecting against, controlling, and providing a public health response to the international spread of disease (*7*). Although the IHR contain articles directed toward several facets of public health security, they can be broadly summarized into 2 main areas: urgent actions to be taken with respect to acutely arising risks to public health and strengthening of national systems and infrastructure (referred to as core capacities). This article provides an overview of selected contributions to these areas made during the past 5 years. It is written from the perspective of the WHO department charged with coordinating implementation of the IHR at WHO global headquarters in Geneva and seeks to identify major achievements and continuing challenges.

## Establishment of National IHR Focal Points

One of the early demonstrations of global commitment to implementation of the IHR has been the successful establishment of National Focal Points (NFPs) in all but 1 of the states parties to the IHR. (States parties to the IHR include all WHO member states, the Holy See [an observer to the World Health Assembly], and Liechtenstein.) NFPs are national centers, not individual persons, that occupy a critical role in conducting the communications aspects of the IHR, within their countries and internationally (*8*). They are responsible for proactively notifying WHO of relevant health events, responding to WHO secretariat requests for event-related information, and ensuring that messages and advice from WHO are disseminated to the relevant actors within the country. Since 2007, NFPs have been increasingly diligent in updating and confirming their contact details to WHO on an annual basis as required by the regulations. NFPs are officially sanctioned to work with WHO on IHR implementation and provide feedback to WHO on country needs and concerns for this task. Staff members who work in NFPs are a major audience for WHO training materials. The engagement of NFPs in the scientific evaluation of the IHR notification procedures has indicated that a high proportion of NFPs had a good understanding of the notification procedures and had accessed WHO training materials on this issue and has indicated that agreement was high in terms of events that must be notified when applying the procedures (*9*). NFPs have access to the contact details of all other NFPs through a password-protected website that enables direct communication among countries at the NFP level. For events that do not require WHO coordination (such as routine tracing of contacts for an infectious disease associated with international travel), such direct communications have been useful.

Not all NFPs are able to function as expected. For example, some contact details fail to work for urgent communications, some NFPs indicate that procedures for round-the-clock communications are not yet established, and delays in responding to requests for event information often occur. Studies have indicated that NFPs know how to assess events under the IHR. Their participation in event-related communications is increasing; however, their role has been primarily providing official and accurate information on events that first gain WHO attention through informal sources such as media reports. Among the reasons identified for such less-than-optimal performance is that some NFPs lack authority or access to the necessary authority, resulting in delays in obtaining clearance for communications. Such lack of authority is also identified as a barrier to the effective intersectoral collaboration that is envisioned as critical to the NFP role within their national situation. Although NFPs generally recognize the value of engaging with government sectors outside the health ministry, they lack the convening power needed to establish solid and reliable linkages.

## Pilot Testing of IHR-Implementation Course

A key WHO objective is to strengthen the human resources available to countries to set up and manage systems for securing global public health under the IHR framework. In partnership with established educational institutions, the WHO secretariat has been pilot testing an IHR-implementation course, which promotes a global harmonized understanding and application of the IHR framework.

The IHR-implementation course is for public health professionals, mainly those belonging to NFPs but also those from other related sectors from national or international organizations in public and private sectors. The course is delivered over 5 months as on-the-job training. The 210 total learning hours consist of 12 weeks of distance learning with tutoring and a 6-week break used to finalize assignments and prepare for the 2-week face-to-face session.

The first 3 pilot IHR-implementation courses have been operated by the WHO Department of Global Capacities, Alert and Response in collaboration with the University of Pretoria, South Africa; Georgetown University Law Center, USA; the University of Geneva, Switzerland; and Institut Bioforce Développement, France. Implementation of the courses involved the contributions of several WHO departments: Food Safety, Zoonoses and Foodborne Diseases; Protection of the Human Environment; Health Action in Crises; and Health Systems and Services. WHO Regional Offices have been mobilized to identify and sponsor participants.

The IHR-implementation courses have been delivered in English to 89 participants from 57 countries in all 6 WHO regions. Post-training evaluation of the first 2 courses conducted in 2011 indicated that the course content was relevant to participants’ work, improved their understanding of IHR, and increased their confidence when dealing with the topic. Competencies developed have been put into practice, and material from the course has been re-used at the national level. The opportunity to engage with peers from other countries during and after the course was considered especially valuable.

In light of the positive evaluation and continuing need, organization of additional courses at the national level is planned. A need to provide the course in languages other than English requires new institutional partners and additional resources. Some of the IHR-implementation course contents are being developed into stand-alone modules for potential integration into other established training opportunities such as field epidemiology training and Masters of Public Health programs.

## Monitoring of Progress of IHR National Core Capacities

One of the most substantial obligations introduced by the IHR is the commitment of states parties to develop, strengthen, and maintain national capacities to identify, investigate, assess, and respond to public health events in their territories and to develop, strengthen, and maintain routine and emergency public health capacities at certain designated points of entry. These obligations were introduced in acknowledgment that effective national systems are the essential underpinning to any global health security and that such systems are the mechanisms needed to prevent many public health events from reaching the level of international significance. The IHR capacities are described in functional terms in Annex 1, and a major milestone toward implementation has been to reach a consensus on the scope and technical components that can be expected to contribute to the required functionality.

For surveillance and response, the capacities are grouped under the following 8 main headings:

- National legislation, policy and financing
- Coordination and NFP communications
- Surveillance
- Response
- Preparedness
- Risk communication
- Human resources
- Laboratory

A range of potential health hazards can fall under the IHR capacity requirements. These hazards have been identified as infectious, zoonotic, food safety, chemical, and radiologic/nuclear.

To help states parties assess their capacity, a monitoring framework was developed. The framework represents a consensus of technical expert views drawn globally from WHO member states, technical institutions, partners, and from within WHO. The framework incorporates current knowledge and concepts that have been successfully used to monitor capacity-development activities. It builds on the experts’ knowledge of current capacities of states parties, existing regional and country strategies for capacity development, and other available resources and tools, particularly other tools used for IHR core capacity assessment by states parties. Using a checklist of 20 indicators, the IHR monitoring process assesses status of implementation in 8 areas of core capacity, development of capacities at points of entry, and development of capacities for the IHR-relevant hazards.

An annual questionnaire is used to collect data on the core capacities; country responses are stored in a secure database at WHO, accessible only to IHR NFPs and the secretariat through use of tools that ensure country confidentiality. The questionnaire is made available in several formats, including through the Internet. To ensure that the full spectrum of relevant hazards is covered, NFPs are advised to lead the process of completing the questionnaire, in close collaboration with officials responsible for the various capacity areas and including other sectors.

Outputs of the monitoring framework include country profiles for all reporting countries and detailed NFP reports on strengths, weakness, and gaps; profiles for the 6 WHO regions; and aggregated global reports for the World Health Assembly. This information has enabled states parties to measure progress and identify where improvements are needed, thereby providing evidence for program planning, recommendations, and decision making. At the global level, this monitoring information is used by the secretariat to comply with the Health Assembly request for an annual report on IHR implementation from WHO, including information provided by states parties and on the secretariat’s activities. Thus, WHO governing bodies can take account of the progress when directing secretariat activities. The analysis also enables better identification of the priority areas toward which the secretariat and other development partners can focus their support to countries.

From a total of 194 states parties, the questionnaire elicited 128 and 156 responses for 2010 and 2011, respectively. Because not all states parties responded to the questionnaire, the reports produced might not completely reflect IHR core capacity development strengths and weaknesses at the regional and global levels. Evaluating implementation status in nonresponding countries is challenging, especially because some of these countries face the greatest implementation difficulties. With the goal of improving the validity and consistency of self-reported data, several multicountry workshops and trainings have been held and standardized data collection and analysis tools have been promoted. Such challenges are also being addressed by identifying several supplementary information sources that might partially reflect national IHR capacities and including such information in an additional report to the 2012 Health Assembly.

The biggest challenge involved in implementing the IHR is ensuring that the IHR core capacities are present in all countries of the world. Ensuring IHR core capacities is also the area in which the IHR have the greatest potential to make a major contribution to world health; as the process approaches a key 5-year milestone on June 15, 2012, all efforts are being refocused on this issue.

## Interagency Collaboration for Public Health at Points of Entry

Although many IHR provisions address international travel and transport and public health activities at points of entry (ports, airports, and ground crossings), these have not been areas in which WHO or many member states had strong preexisting programs. Attention has therefore been focused on leveraging interagency and multisectoral collaboration at all levels to achieve the public health objectives. For example, the Cooperative Arrangement for the Prevention of Spread of Communicable Disease through Air Travel project (*10*) is an initiative of the WHO sister agency the International Civil Aviation Organization, through which countries can receive support for realizing IHR objectives relating to air travel. Other collaborations include the International Tourism Response Network (*11*), regional networks such as the Risk Assessment Guidance for Infectious Diseases Transmitted on Aircraft project (initiated by the European Centre for Disease Prevention and Control) (*12*), and the European Commission ship sanitation training network project (*13*). To facilitate information sharing and coordination among authorities responsible for health measures and development of IHR core capacities at points of entry, WHO supports a specialized network for ports, airports, and ground crossings: the PAGnet (*14*). During the 2011 nuclear accident in Japan, the 2010–11 cholera epidemic in Haiti, and the 2009 influenza A (H1N1) pandemic, PAGnet offered a communication platform to public health officials at points of entry around the world, facilitating timely information sharing on response measures that helped avoid overreaction and unnecessary barriers to international travel and trade.

Although assessments have shown many IHR capacities at certain points of entry in several countries, countries differ widely in the levels of capacity, the allocation of responsibilities, and the priority given to this area of public health. This heterogeneity makes it more difficult to provide guidance and advice that is relevant to the national and local contexts of all ports, airports, and ground crossings around the world. Private industry and commercial organizations, which involve a variety of governmental sectors in addition to health, are key actors for the implementation of IHR provisions affecting travel and transportation. WHO must use its convening power, its neutrality, and its focus on public health objectives to help the disparate actors reach consensus.

## Pandemic Influenza and Convening of the Emergency Committee

Around the world, many IHR provisions are used daily. Thus far, however, the full range of provisions relating to global emergencies have been applied to only 1 event: the 2009–2010 influenza pandemic. The IHR define a category of events with the term “public health emergency of international concern.” The WHO director-general follows defined procedures to determine which events are so characterized. The key practical outcomes of such a determination are the provision of relevant information to all states parties, the convening of an IHR Emergency Committee to advise the director-general regarding the event, and the issuance of IHR temporary recommendations.

The first IHR Emergency Committee was convened on April 25, 2009, to advise the WHO director-general about the determination of the first public health emergency of international concern under the IHR. That this first meeting of the Emergency Committee took place by teleconference within 48 hours of the decision to convene it demonstrated that the procedures established by the IHR could work in practice. The continued work of this committee, providing advice to the director-general for more than a year, demonstrates the commitment of its members to support the governments of the world and WHO in their responses to the emergency. During the influenza pandemic, the NFP network developed much-needed momentum and provided early information and situation updates as the virus was identified around the world. The WHO secretariat was able to provide updates, announcements, and advice to countries through the event information site for NFPs with timing that was coordinated with its provision of public information.

The duration of the public health emergency of international concern posed several challenges for the procedures established for IHR implementation. For example, the decision to protect the impartiality of the advice given by members of the IHR Emergency Committee (by not publishing their names until after their work was completed) was not helpful when their work went on for more than a year and was under intense media speculation. Also, the rules adopted for temporary recommendations were designed to allow them for only a limited amount of time, which was just barely compatible with the pandemic experience. The IHR did not prevent several countries from applying restrictive travel- and trade-associated measures not recommended by WHO, although several such measures were discontinued or modified after communication with the WHO secretariat. The IHR Review Committee was concerned by the restrictive measures and provided recommendations on how they can be more effectively addressed (*15*).

## Establishment of External IHR Review

The potential to learn lessons from the 2009–2010 pandemic influenza experience and the need to address public concerns regarding the WHO response led to the establishment of the first IHR Review Committee. The remit of this committee was expanded (by the WHO Executive Board from a periodic review of the functioning of the IHR, as required under IHR Article 54) to include an independent, external review of the international response to pandemic influenza. Although the secretariat provided administrative and logistic support, the committee, under the chairmanship of Harvey Fineberg, enjoyed complete autonomy in interpreting their mandate, defining their methods of work, and identifying their evidence. In doing so, they followed the requirements of the IHR in ensuring states parties the opportunity to observe and engage in formal committee meetings. After more than a year, the committee delivered its final report to the 64th Health Assembly, at which the approach taken was commended and the recommendations were endorsed by the member states. Despite findings that WHO faced systemic difficulties and some shortcomings in addressing the influenza pandemic, the committee concluded that the actions taken were motivated by public health concerns and found no evidence of misconduct. The 15 recommendations in this report have gone on to form a major component of the biennial work plans of the relevant WHO departments.

The exhaustive work of the IHR Review Committee made heavy demands on the time of its expert members and on WHO resources. WHO should take advantage of the exceptional opportunity to learn from this analysis of the pandemic experience.

The IHR allow review committees to give advice broadly on the functioning of the regulations, and it can be foreseen that in future years, committees will need to be convened with markedly different tasks, for example, advising on the granting of a second round of extensions to the core capacity time frame. At such time, the working methods of such a future review committee will need to be reassessed to fit with its mandated task.

## Conclusions

The IHR are a legal tool designed to contribute to the achievement of public health goals, in which success is seen and measured in improvements to public health rather than adherence to any particular article of the document. At the same time, given the large number of initiatives for and influences on public health outcomes, it will always be hard to tease out and identify the specific contributions of such an instrument to global health. This article indicates some of the direct effects that IHR implementation is having on public health practice. Where states and WHO are building on preexisting programs, the IHR have boosted continuing commitment and momentum. An example at the international level is the WHO program for management of acute public health events; an example at the country level is the program to strengthen capacity in public health laboratories. In addition to boosting existing programs, some new activities relating to IHR provisions have been successfully established, such as the NFP network and the Emergency Committee.

The lessons and experience of the past 5 years need to be drawn upon to provide improved direction for the future. The member state–driven negotiations provide a legacy of ownership and commitment from countries, which continues to be evident in the nature and number of interventions concerning IHR during meetings of WHO governing bodies. As we approach the 5-year target date of June 2012, the immediate challenge is for WHO and the states parties to live up to the intention of the IHR national core capacity requirements and to make the best use of the opportunity for countries to continue their efforts beyond that date as anticipated under the extension procedure provided by the IHR.
